# The ‘Ready Method’ in Cases of Narcotic Poisoning

**Published:** 1857-05

**Authors:** 


### *The 'Ready Method' in Cases of Narcotic Poisoning.*
By Marshall Hall, M. D.

Many years ago a lady drove to my door in the utmost consternation, having shortly before swallowed an ounce and a half of Battley's solution of opium, in mistake for a senna draught.

I instantly applied the stomach pump with my own hands, and her life was saved.

I have often asked myself the question: What should I have done had I not had the stomach pump in all readiness, and had not been able to induce vomiting? Until this hour, I knew not how satisfactorily to answer this fearful question.

There are two stages in narcotic poisoning, in each of which the 'Ready Method' is, in the absence of the stomach pump, our *hope.*

The *first* is, that in which our object is to remove the poison from the stomach by the induction of *vomiting*, but in which, from the degree of narcotism, all ordinary remedies fail.

The *second*, that in which our object and hope are, to continue respiration *until* the elimination of the poison from the system may be accomplished.

In the former case, the patient should be laid on a table, with the head projecting beyond its edge, if possible; if not, on the floor; and being placed on the *side*, the finger of one person is to be introduced into the *fauces*, whilst the body is repeatedly and briskly rolled into the *prone* position by another.

If there be the slightest degree ('*scintillula*') of excito-motor power remaining, the *cardia*, already somewhat relaxed, perhaps, from torpor, will be still further relaxed, physiologically, whilst the *glottis*, the safety-valve of the trachea, is closed, and the *thorax* and *abdomen* being compressed by a force equal to the superincumbent weight of the body, to which further force may be added by means of pressure made along the spine, *mechanical* vomiting will be produced, and the poison expelled.

This desirable effect will be produced in cases in which the narcotic torpor is too great to admit of exciting the very complex act of *physiological* vomiting.

But now let us suppose that the narcotism is too deep for the success of this manœuvre—that the *second* case is before us. Then our hope consists in continuing respiratory movements until the poison is eliminated from the blood and the general system. In one word, our hope is in the 'Ready Method,' such as I have recommended it for asphyxia!

I suppose that volition has ceased, and that the patient can no longer be made to move or walk about; that all good physiological respiration has ceased, or is about to cease; then, one hope still remains—postural respiration—and the other measures comprised in the 'Ready Method in Asphyxia;' and I need not say how long and perseveringly this method should be continued.—*Lancet*, Jan. 17, 1857.

---

